# The Use of Hybrid Genetic Algorithm in the Kinetic Analysis of Thermal Decomposition of [Ni(C_2_H_8_N_2_)_3_](ClO_4_)_2_ with Overlapping Stages

**DOI:** 10.3390/ma16010090

**Published:** 2022-12-22

**Authors:** Kirill A. Dmitruk, Oksana V. Komova, Alexander A. Paletsky, Andrey G. Shmakov, Svetlana A. Mukha, Vladislav R. Butenko, Alena A. Pochtar, Olga V. Netskina

**Affiliations:** 1 Boreskov Institute of Catalysis SB RAS, 5 Akademika Lavrentieva Ave., Novosibirsk 630090, Russia; 2 Department of Natural Sciences, Novosibirsk State University, 1 Pirogova Str., Novosibirsk 630090, Russia; 3 Siberian Branch of Russian Academy of Sciences, 17 Akademika Lavrentieva Ave., Novosibirsk 630090, Russia; 4 Voevodsky Institute of Chemical Kinetics and Combustion SB RAS, 3 Institutskaya Str., Novosibirsk 630090, Russia

**Keywords:** thermal analysis, dynamic mass-spectral thermal analysis, two-step kinetics, Coats–Redfern kinetics, tris(ethylenediamine)nickel(II) perchlorate

## Abstract

This work describes the mathematical modeling of the thermal decomposition of the complex compound [Ni(En)_3_](ClO_4_)_2_ (En = C_2_H_8_N_2_ = ethylenediamine) in an inert atmosphere under non-isothermal conditions. This process is characterized by several simultaneous and intense stages: elimination of ethylenediamine from the nickel coordination sphere, decomposition of perchlorate anions, and explosive-like oxidation of free or bound ethylenediamine. These stages overlap and merge into a one step on the differential thermogravimetric curve. Typically, this curve is modeled as a one-stage process during kinetic analysis. In this paper, for the first time, the data from the dynamic mass-spectral thermal analysis and thermogravimetric analysis were modeled using the hybrid genetic algorithm, and the results were compared. A two-stage scheme of [Ni(En)_3_](ClO_4_)_2_ thermolysis was proposed and the kinetic parameters for each stage were obtained. It was shown that the decomposition of [Ni(En)_3_](ClO_4_)_2_ begins with the elimination of one molecule of ethylenediamine (stage A), then the perchlorate anions quickly decompose with the evolution of oxygen (stage B). We believe that the resulting ${\mathrm{ClO}}_{4-x}^{-}$ (x = 1–3), as stronger oxidizing agents, instantly start an explosive-like exothermic process of ethylenediamine oxidation (stage B).

## 1. Introduction

Thermal analysis is a widely used method of studying the thermal properties of different substances and materials. The predictions and mathematical descriptions of the decomposition/combustion processes under the action of temperature are essential for solving the important practical problems: fire propagation [1,2,3,4], self-propagating high-temperature synthesis of materials [5,6,7], waste disposal [8,9,10], development of new fuels [11], etc.

The estimation of Arrhenius kinetic parameters of solid-state decomposition processes based on the linearization of thermal analysis data (such as Coats–Redfern, Freeman–Carrol, Horowitz–Metzger techniques) is widely used and does not require specialized software. The linearized Coats–Redfern equation (Equation (5), Section 2.3) is applied for describing the single-stage processes, multi-stage processes with one limiting stage, and multi-stage processes with non-overlapping peaks on the differential thermogravimetric (DTG) curve [9,12,13,14,15,16]. Unfortunately, this method has low accuracy and does not describe even a single-stage thermolysis process [17,18]. This is clearly shown in [18] where the authors simulated single-stage and two-stage thermolysis processes with different mechanisms and analyzed their Coats–Redfern linear approximations. In the case of single-stage models with different kinetics, the linear plots with similar correlation coefficients were observed. The thermogravimetric data for two-stage models with overlapping stages were not linearized.

Modeling of solid-state processes becomes much more complicated in the case of multi-stage thermal decomposition, characterized by overlapping peaks on the DTG curve [19]. It was found that in this case the linear Coats–Redfern equation (Equation (5), Section 2.3) cannot be reliably applied [18,20]. In order to calculate the kinetic parameters (A, E, n) and establish the kinetic models of every stage of the processes, DTG can be deconvoluted. In practice, the Gauss, Lorentz, or Fraser–Suzuki functions are applied [8,21,22,23,24]. In this case, the use of computer calculations is inevitable. Currently, in addition to deconvolution, there are several iterative computer algorithms for numerically solving the systems of kinetic equations and finding many kinetic parameters of thermal decomposition processes using inverse modeling. These algorithms search for the global minimum of an objective function, which reflects the difference between the set of kinetic parameters and experimental data [2,10]. In such cases, the optimized value of the objective function (F) or its logarithm (lgF) are used instead of determination or correlation coefficients to compare the different solutions.

Thus, the evolutionary algorithms, and in particular, the genetic algorithm (GA) (described in more detail in Section 2.4) has been used recently to overcome such problems [2,10,25,26,27,28,29]. However, GA can take an unreasonable amount of time and resources to find the exact solution, and the solutions acquired using this algorithm might have low repeatability in subsequent runs of GA. In addition, an increase in the number of parameters associated with the complication of the mechanism of the thermolysis process, as well as setting wide numerical boundaries for their search, increases the resource intensity of computer calculations. Despite the fact that the use of GA to describe complex processes of thermal decomposition is still not widespread, the relevance of two directions is already obvious: (1) reducing the time required to search for kinetic parameters and (2) searching for ways to refine their values.

That is why, when describing the multi-stage pyrolysis process of the medium-density fiberboard a more optimized variant of GA was proposed. Its application has been shown to improve the speed and accuracy of calculations. In this modeling, the number of stages was established from the number of inflection points on the DTG curve, and then the kinetic parameters A and E were estimated using the Kissinger method to significantly narrow down the search range for GA [30]. In [10] more refined and reliable results were obtained when using the hybrid genetic algorithm (hybrid GA) to model the agricultural waste decomposition processes. In the first stage, GA was applied to find the kinetic parameters that are likely to be close to the global minimum of the functional. The second step was the application of the least squares fitting to refine the results acquired by GA. The non-linear fitting algorithm was also successfully used in the modeling of the pyrolysis of pine branches as a second algorithm in the hybrid GA [27].

On the other hand, in comparison with the numerical integration of the differential Equation (1) or (2) (Section 2.3), using the nonlinear Coats–Redfern approximation (Equation (4), Section 2.3) in GA for the modeling of the multi-stage thermolysis processes can reduce the amount of time required for computation. In the literature, the Coats–Redfern approximation (Equation (4), Section 2.3) was successfully used in GA for the modeling of the three-stage thermolysis of ammonium pentaborate octahydrate [31], and the two-stage thermolysis of ulexite [32]. It was shown that the GA with the Coats–Redfern approximation (Equation (4), Section 2.3) is a more effective approach to obtaining kinetic parameters than its linearized form (Equation (5), Section 2.3) because the approximation 2RT/E << 1, which reduces the calculation accuracy, was not applied.

In this paper, we will continue to study the possibilities of using the nonlinear Coats–Redfern approximation (Equation (4), Section 2.3) in GA for the modeling of the thermal analysis data for complex compounds [Ni(En)_3_](ClO_4_)_2_ (En = ethylenediamine C_2_H_8_N_2_) in an inert atmosphere under non-isothermal conditions. Despite the fact that this energetic compound containing a fuel-oxidizer system is well known [33], the modeling of its thermal decomposition has not been discussed in the literature. Based on the literature [34,35], it can be assumed that the gasification of [Ni(En)_3_](ClO_4_)_2_ should be described by multiple stages associated with the removal of ligands from the coordination sphere of nickel, the decomposition of perchlorate anions and the oxidative degradation of the ligand. However, the observed differential mass loss curve (DTG curve) and the differential heating curve (or the differential scanning calorimetry (DSC) curve) consist of one narrow peak, which is typical for explosive-like thermal decomposition of energetic compounds [34,36,37]. In contrast to [31,32], in this work, hybrid GA with the Nelder-Mead (NM) simplex algorithm [38] as the second step will be used to find the solution of the Equation (4) (Section 2.3). For the first time, the modeling of the thermogravimetric data for [Ni(En)_3_](ClO_4_)_2_ decomposition (the heating rate of 5 °C·min^−1^) will be compared to the modeling of its gasification data obtained from dynamic mass-spectral thermal analysis (DMSTA). Joint consideration of the results of these independent thermal analysis methods allowed the proposed model to be verified without the need for TG/DSC experiments at different heating rates [39]. The proposed model predicts the DTG curves obtained at a lower heating rate of 2.5 °C·min^−1^ well, confirming the applicability of the applied modeling approach. As a result, for the explosive-like thermal decomposition of [Ni(En)_3_](ClO_4_)_2_, a two-stage scheme will be proposed and the kinetic parameters of each stage will be calculated. Density functional theory (DFT) calculations were used to support the obtained results.

## 2. Materials and Methods

### 2.1. Synthesis of [Ni(En)_3_](ClO_4_)_2_ and Its Characterization

For the synthesis of [Ni(En)_3_](ClO_4_)_2_ complex, 0.02 mole of Ni(ClO_4_)_2_∙6H_2_O (98%,TU 6-09-02-118-86) were dissolved in 10 mL of ethanol. The obtained solution was cooled down to 1 °C. Under vigorous stirring and cooling in an ice bath, this solution was added to the solution of 0.08 mole of ethylenediamine (99.9%, CAS RN 107-15-3) in 10 mL of ethanol. At the same time, the temperature of the reaction mixture increased to 42 °C. An instantaneous appearance of purple precipitate was observed. The mixture was stirred for 7 min, while its temperature dropped to 5 °C. Then the complex precipitate was filtered, washed with a small amount of cold water and ethanol, dried in a vacuum oven, then over P_2_O_5_. The product yield was 92%. The theoretical content of Ni, C, H, N, and Cl in the [Ni(En)_3_](ClO_4_)_2_ complex should match 13.40 wt%, 16.46 wt%, 5.52 wt%, 19.19 wt%, and 16.19 wt%, respectively. The experimental Ni (13.05 wt%), C (16.37 wt%), H (5.77 wt%), N (19.31 wt%), and Cl (15.72 wt%) contents are in good agreement with the theoretical ones. The infrared spectrum of the synthesized sample is shown in the Supplementary Materials (Figure S1). The characteristic absorption bands (cm^−1^) are 3350 (s), 3300 (s), 3180 (w), 2947 (m), 2893 (m), 1577 (m), 1463 (m), 1398 (w), 1326 (w), 1281 (m), 1064 (s), 1012 (s), 653 (m), 620 (s), 505 (m), 497 (m), 477 (m), 402 (w), 325 (w).

The Ni content was determined by inductively coupled plasma atomic emission spectrometry on an Optima 4300 DV instrument (PerkinElmer, Waltham, MA, USA). The C, H, and N contents were determined on an automatic CHNS analyzer EURO EA 3000 (Euro Vector S.p.A., Castellanza, Italy). The samples (0.5–2 mg) were combusted in a vertical reactor in the dynamic regime at 1050 °C in a flow of He with addition of O_2_. The chlorine content was determined by the standard method after combustion of the sample according to the Schöniger oxygen-flask technique followed by mercurimetric titration of chloride anions in the presence of diphenylcarbazone [40].

The attenuated total reflection infrared spectroscopy (ATR FTIR) was performed on an Agilent Cary 600 (Agilent Technologies, Santa Clara, CA, USA) spectrometer equipped with a Gladi ATR (PIKE Technologies, Madison, WI, USA) attachment in the range 300–4000 cm^−1^ without a pretreatment of the sample.

### 2.2. Study of Thermal Properties of [Ni(En)_3_](ClO_4_)_2_

The thermal analysis was performed on a Netzsch STA 449 C Jupiter instrument equipped with a DSC/TG holder (NETZSCH, Selb, Germany) in the temperature range 20–500 °C in the corundum crucible under a flow of helium (30 mL·min^−1^). The heating rate (β) of the samples was 2.5 or 5 °C·min^−1^, and the weight of the samples was 5 mg.

The gases evolved during the thermal decomposition of [Ni(En)_3_](ClO_4_)_2_ were analyzed by the dynamic mass-spectral thermal analysis (DMSTA) method, using a time-of-flight mass spectrometer with a molecular beam sampling system MSCh-4 (Plant Of Scientific Instrumentation, Sumy, USSR) under a flow of Ar (5 mL·min^−1^). The average heating rate was 15 °C·s^−1^. The sample weight was 5 mg. The delay between measurements was 0.04 s. The identification of mass spectral signals was carried out using the mass spectra of individual substances from the NIST database.

### 2.3. Kinetic Theory of Solid-State Decomposition

It is well known that the kinetics of solid-state processes pose a complex problem [41,42,43,44]. Most solid-state decomposition models are based on the assumption that the reaction rate can be written as:

(1) $$\frac{d\alpha }{\mathrm{dt}}={\mathrm{Ae}}^{-\frac{E}{\mathrm{RT}}}f\left(\alpha \right),$$

where α—conversion fraction; f(α)—reaction model depending on α (Table 1); t—time; A—preexponential factor; E—activation energy; R—gas constant; T—temperature of the sample [3,25,43,45,46].

In non-isothermal conditions with a linear heating rate β the temperature depends on time as T = T_0_ + βt and the non-isothermal reaction rate can be written as:

(2) $$\frac{d\alpha }{\mathrm{dT}}=\frac{A}{\beta }e^{-\frac{E}{\mathrm{RT}}}f\left(\alpha \right)$$

After separating the variables and integration, the following equation can be produced:

(3) $$g\left(\alpha \right)\equiv \int_{0}^{\alpha }\frac{d\alpha }{f\left(\alpha \right)}=\frac{A}{\beta }\int_{0}^{T}e^{-\frac{E}{\mathrm{RT}}}\mathrm{dT},$$

where g(α) is the integral reaction model (Table 1).

The integral on the right side of Equation (3) cannot be found analytically, however, there are several approximate solutions of the integral [44], out of which the Coats–Redfern approximation is commonly used [45]. The result of the Coats–Redfern approximation is Equation (4):

(4) $$g\left(\alpha \right)=\frac{{\mathrm{ART}}^{2}}{\beta E}\left(1-\frac{2\mathrm{RT}}{E}\right)\exp\left(-\frac{E}{\mathrm{RT}}\right)$$

Taking into account that usually $\frac{2\mathrm{RT}}{E}$ << 1, its linear approximation is widely used in practice:

(5) $$\ln\left(\frac{g\left(\alpha \right)}{T^{2}}\right)=\ln\left(\frac{\mathrm{AR}}{\beta E}\right)-\frac{E}{\mathrm{RT}}$$

Fitting Equation (5) with the varying g(α) (Table 1) to experimental data and comparing the applicability of the different reaction models via correlation coefficient or determination coefficient allows choosing the best mathematical model to describe the kinetics of the decomposition process. 

### 2.4. Modeling of the Experimental Data of [Ni(En)_3_](ClO_4_)_2_ Thermolysis

For some tasks of searching for the global minimum of the objective function, simple algorithms may be used, such as random search or the Quasi–Newton method [47], but there are problems when calculating the kinetics even of a single-stage process with two calculated parameters due to a high number of local minima (Figure S2). In addition, the modeling of the multi-step thermolysis process is also complicated by the high dimensionality. Therefore, as mentioned earlier, various evolutionary algorithms are used to overcome such problems. One of the most commonly used algorithms is the genetic algorithm (GA). It is also one of the most resource-saving evolutionary algorithms, and is characterized by high accuracy [2]. 

The GA is based on the principles of evolution—initially a random population is created, where each individual is a vector of kinetic parameters within the established limits. The individuals that correspond to the lowest value of the function have higher fitness, so they are combined to form a new population corresponding to the next generation. Within this algorithm, local minima are avoided through mutations—random changes in parameters. The assurance that the GA does not move away from the solution in subsequent generations is the selection of elites—the fittest individuals that remain unchanged in the next generation.

In this work during the modeling of the DTG and DMSTA data the following well-known objective function was used:

(6) $$F=\frac{1}{N}\overset{N}{\underset{i}{\sum }}\left(\gamma_{1}{\left(\alpha_{i}^{\mathrm{theor}}-\alpha_{i}^{\exp}\right)}^{2}+\gamma_{2}{\left({\frac{{d\alpha }^{\mathrm{theor}}}{\mathrm{dt}}}_{i}-{\frac{{d\alpha }^{\exp}}{\mathrm{dt}}}_{i}\right)}^{2}\right),$$

where N—number of experimental points; $\alpha_{i}^{\mathrm{theor}}$ and $\alpha_{i}^{\exp}$—simulated and experimental conversion fractions at the temperature i; ${\frac{{d\alpha }^{\mathrm{theor}}}{\mathrm{dt}}}_{i}$ and ${\frac{{d\alpha }^{\exp}}{\mathrm{dt}}}_{i}$—simulated and experimental reaction rates at the temperature i; γ_1_ and γ_2_—weight of the integral and the differential halves of the functional [2]. In all cases γ_1_ = 1, but γ_2_ = 1/x, where x is the maximum of $\frac{{d\alpha }^{\exp}}{\mathrm{dt}}$ in the DTG curve. This was done due to the fact that the decomposition rates had much smaller values than the conversion fractions because of their different dimensions. This allowed us to increase the accuracy of the kinetic parameters and decrease the time needed for the algorithm to run (Figure S3). In the applied in this work version of the hybrid GA, at the second stage, the Nelder–Mead (NM) simplex algorithm provided in the MATLAB software package (Version R2018a, The Mathworks, Inc., Natick, MA, USA) was used to refine the results. When modeling the DTG curve as a single- or two-stage process, kinetic models 1–11 and 12 with *n* from 1 to 3.5 with the step of 0.5 (Table 1) were used. It should be noted that models 7 and 12 with *n* = 0.5 are equivalent. Simulated curves were built using the Coats–Redfern approximation (Equation (4)).

The GA in this work was used as provided in the MATLAB software. Each population consisted of 100 individuals—vectors of parameters (lgA_1_, lgA_2_, E_1_, E_2_, r), where lgA_1_ and lgA_2_ are the logarithms of the preexponential factor of the first and the second stages, respectively; E_1_ and E_2_ are the activation energies of the first and the second stages, respectively; r is the contribution of the first reaction to the total process. The “max generation” setting, i.e., the maximum number of cycles in the GA, was set to 100. The number of individuals created through mutation in each population was 50%, and a Gaussian mutation function was used to control the standard deviation with each generation. The “scale” parameter was set to 0.1 to ensure a small standard deviation in order to reduce the probability of missing the convergent solution during mutations. The linear “shrink” parameter, which controls the rate at which the standard deviation decreases with each generation, was set to 0.5. A value of the “shrink” parameter closer to 1 is more effective for finding convergent solutions [48], however, in the case of the hybrid GA the acquired results do not have to converge because of the refinement step using the second algorithm. “Function tolerance”, i.e., the value of the variation of the objective functional (Equation (6)), at which the solution was considered to converge, was set to 10^−8^ for the GA and to 10^−12^ for the subsequent refinement with the NM algorithm, which ensured the convergence of the refined solution. Other parameters were assigned to the default settings of the GA within the MATLAB framework and did not require any changes.

A wide range was set for the initial population (lgA from 10 to 30, E from 200 to 400 kJ/mol), which increases the probability of some individuals to be near the global minimum of the functional. Additionally, the unconstrained variant of GA was used in this work, which avoids errors in case of incorrect assumptions about the search range. Each run of the hybrid GA was repeated 4 times to ensure that the acquired solutions are repeatable.

The DMSTA data was modeled by the same method, however, the heating rate β was not constant during the high-speed decomposition, which means Equation (3) and its approximations could not be applied in this case. Thus, the simulated DMSTA curves were built using the numerical integration of Equation (1).

The molar ratio of the evolved gases during the DMSTA was determined using the calibration coefficients for ethylenediamine (K_En_ = 0.65) and oxygen (K_O2_ = 1.1) in Ar according to the equation:

(7) $$\frac{\chi_{\mathrm{En}}}{\chi_{O2}}=\frac{\int_{t_{0}}^{t_{\mathrm{fin}}}\frac{{\mathrm{dI}}_{\mathrm{En}}}{\mathrm{dt}}\mathrm{dt}}{\int_{t_{0}}^{t_{\mathrm{fin}}}\frac{{\mathrm{dI}}_{O2}}{\mathrm{dt}}\mathrm{dt}}\cdot \frac{K_{O2}}{K_{\mathrm{En}}},$$

where $\chi_{\mathrm{En}}$ and $\chi_{O2}$—molar fractions of ethylenediamine and oxygen in the evolved gases; t_0_ and t_fin_—time of the start and the end of the evolution of gases; dIEndt and dIO2dt—the rates of mass peak intensity changes for ethylenediamine (*m*/*z* = 30) and oxygen (*m*/*z* = 32). Calibration coefficients were determined by methods described in the literature [49,50]. For the O_2_ calibration, 10% of O_2_ was added to the argon flow; for the ethylenediamine calibration, ethylenediamine (99.9%, CAS RN 107-15-3) was vaporized in the argon flow.

### 2.5. Density Functional Theory Calculations

The molecular modeling calculations were performed using the Orca program (Version 5.0.1) [51]. Geometry optimization of complexes in the ground state was obtained employing density functional theory (DFT) at the GGA/PBE level with calculations using the B3LYP exchange-correlation functional [52] and 6-311++G(d,p) basis set, which has performed well for Ni(II) complexes [53,54]. To achieve a more accurate description of the charge distribution in compounds, additional diffuse functions were used, since it was recognized that the addition of such functions is necessary for the correct description of charged systems [55]. Ni(II) complexes are open-shell systems, so all calculations were based on the UHF method.

The binding energy of the ligand with the complex (E_b_) was calculated as follows:

(8) $$E_{b}\left({\left[\mathrm{Ni}{\left(\mathrm{En}\right)}_{n}\right]}^{2+}\right)=E\left({\left[\mathrm{Ni}{\left(\mathrm{En}\right)}_{n}\right]}^{2+}\right)-E\left({\mathrm{Ni}}^{2+}\right)-\mathrm{nE}\left(\mathrm{En}\right)$$

The energy of the transition state was estimated using the NEB-CI module with 6-311++G(d,p) basis set for Ni^2+^ atom and a Pople 6-31G(d,p) basis set [56] for all of the other atoms. Grimme’s D2 dispersion correction was also applied [57].

## 3. Results

### 3.1. Reliability of the Proposed Modeling

According to results obtained in [18], the linearization employing the Coats–Redfern method (Equation (5)) cannot reliably determine the correct kinetic model even in the case of single-stage processes. At the same time, all of the plots for all kinetic models are not linear in the case of the processes with two overlapping stages, which makes this method invalid.

**Figure 1:**
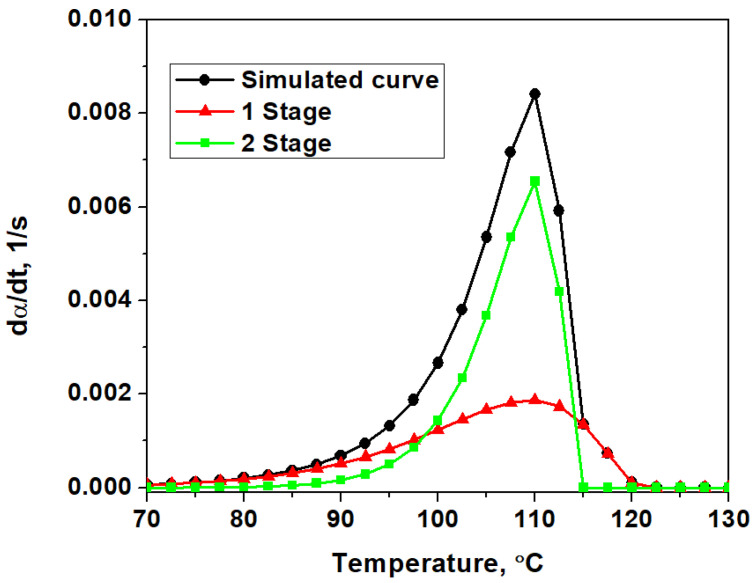
Simulated two-stage DTG curve built using the kinetic parameters from Table 2.

In this case, the applicability of the used hybrid GA with Equation (4) was studied by applying it to a simulated DTG curve built via numerical integration of Equation (2) (Figure 1), characterized by two simultaneous stages with the mechanisms 10 and 8 (Table 2, simulated curve). In the search for the kinetic parameters of the simulated curve, all of the possible combinations of models of Table 1 were tested. Every run of the algorithm converged on the same result. The optimized values of the objective function (Equation (6)) for every model combination are presented in Supplementary Materials (Table S1). The parameters for the original simulated curve along with the best and the second-best fitting predicted curves are shown in Table 2.

The best fitting curve, i.e., the model that has the lowest value F (lgF = −10.82), correctly predicts the kinetic parameters and the mechanism of the simulated reaction. It should also be noted that the second-best prediction has a considerably higher value F (lgF = −8.18) than the first option, which allows us to easily tell that it is less correct, but it still accurately predicts one step and its fraction. Thus, the correct kinetic parameters can be reliably found for two-stage simulated curves using the hybrid GA, which is able to overcome the difficulties experienced when using the linear approximation method (Equation (5)). Therefore, it is possible to use this algorithm to model the experimental data of thermal analysis with a two-stage mechanism.

### 3.2. The modeling of [Ni(En)_3_](ClO_4_)_2_ Thermolysis

#### 3.2.1. Dynamic Gas Evolution Mass-Spectrometry

**Figure 2:**
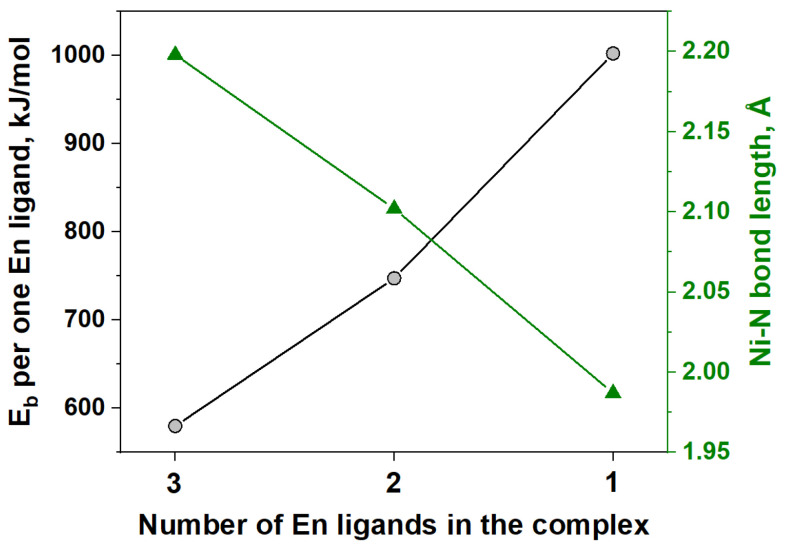
Ni-En bond energies (E_b_) and Ni-N bond lengths for [Ni(En)_n_]^2+^, where *n* = 1, 2, 3, calculated using DFT.

It is known from the literature [34,35,58] that the thermal decomposition of ethylenediamine complexes of transitional metals with non-oxidizing anions (Cl^−^, Br^−^) is characterized by a step-by-step process of elimination of ethylenediamine from the coordination sphere of metal. In our case, the perchlorate anions are present in [Ni(En)_3_](ClO_4_)_2_, which can decompose and/or oxidize the ethylenediamine. Indeed, numerous results on the thermal decomposition of different complexes that contain organic ligands as fuels and anions (ClO_4_^−^, NO_3_^−^) as oxidizing agents are discussed in the literature, the heating of which results in an intense exothermic process of their gasification [35,36,37,59,60,61,62,63,64]. At the same time, it should be noted that the DFT calculations (Figure 2) confirm that the Ni-En bonding energy (E_b_) is relatively low for [Ni(En)_3_]^2+^ (E_b_ = 580 kJ/mol), and consequent removal of En ligands shortens and strengthens the bonds of the remaining ones (E_b_ = 748 kJ/mol and E_b_ = 1002 kJ/mol for [Ni(En)_2_]^2+^ and [Ni(En)]^2+^, respectively). Additionally, [Ni(En)_2_](ClO_4_)_2_ is a known compound that can be prepared separately [33]. Thus, the removal of ethylenediamine ligand is expected in the case of [Ni(En)_3_](ClO_4_)_2_ as well.

**Figure 3:**
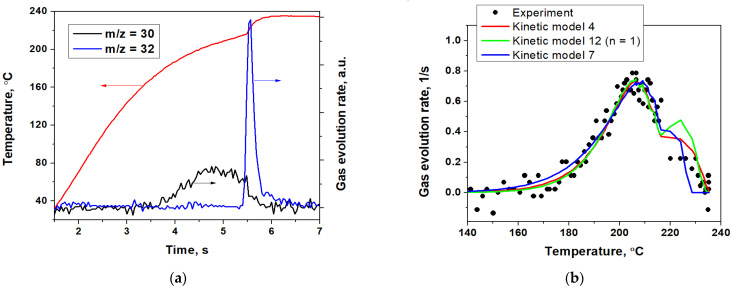
DMSTA results: (**a**) the evolution of gases with *m*/*z* = 30 and 32 and (**b**) the modeling of the evolution of the gas with *m*/*z* = 30 using the hybrid GA.

Therefore, firstly, it is important to know whether the process of ligand elimination without its oxidation occurs in the case of [Ni(En)_3_](ClO_4_)_3_ complex thermolysis. The dynamic mass-spectral thermal analysis (DMSTA) method was utilized for this purpose. The DMSTA curves of evolved gases (Figure 3a) show that under the conditions of the high heating rate (~15 °C/s) the evolution of the gas with *m*/*z* of 30 begins at 180 °C. Since NO evolution is unlikely to occur in such high concentrations when the decomposition of perchlorate anions [65] has not yet begun, we believe that this is caused by the emission of ethylenediamine rather than nitric oxide. In addition, the exothermic effect characteristic of redox transformations of organic compounds [11,66] is not observed in this stage of gas evolution. The intensity of *m*/*z* = 30 reaches its maximum at 205 °C, and at 215 °C an intense evolution of oxygen begins, accompanied by an increase in heating rate from ~15 °C/s to ~50 °C/s due to the exothermic effect of the chemical reactions. It should be noted that the observed exothermic effect can be attributed both to the perchlorate decomposition [65,67] and to the redox transformations in the “ethylenediamine-perchlorate” system [36]. The increase in the heating rate causes an increase in the ethylenediamine evolution rate, and at 235 °C the process stops.

The evolution of the gas with *m*/*z* = 30 (Figure 3b), which we attribute to ethylenediamine, was modeled using the hybrid GA as a one-stage process using the kinetic models 1–11 and 12 with *n* ranging from 1 to 3.5 with the step of 0.5 (Table 1) in the temperature range from 140 to 240 °C. All modeling results are shown in Table S2. The five of the fittest results characterized by the lowest objective function value F (Equation (6)) are shown in Table 3. In this case, due to fluctuations in the heating rate, the numerical integration of Equation (1) was used, since the Coats–Redfern approximation (Equation (4)) is only applicable in the case of a linear heating rate. The evolution of the oxygen gas with *m*/*z* = 32 was not modeled due to the small number of experimental points in the temperature region of its evolution.

As can be seen from Table 3, kinetic model 4 is the fittest, but its value of lgF is very close to the results for other kinetic models. At the same time, the main differences between different kinetic models are observed in the temperature range of 215–235 °C, i.e., in the region where the heating rate increases due to the occurrence of the second stage characterized by the exothermic effect (Figure 3a). The evolution curve of the gas with *m*/*z* = 30 and the simulated curves (Figure 3b) have a high-temperature shoulder, which we believe is associated with the increase in the heating rate, which increases the rate of the ethylenediamine elimination from the [Ni(En)_3_](ClO_4_)_2_ complex. On the other hand, the decomposition of perchlorate ions occurs in the same temperature region, and it can be assumed that there is a redox interaction of ethylenediamine with perchlorate or its decomposition products. In such a case, a decrease in the content of ethylenediamine released into the gas phase is likely. Thus, the accuracy of the modeling in the region of 215–235 °C is significantly reduced. Therefore, at the next step, for the comparison of the constructed models of the ethylenediamine evolution into the gas phase, the region up to 216 °C was chosen. The selection of the kinetic model was made on the basis of the comparison of lgF values (Table 3). The kinetic model 7 (which is equivalent to the kinetic model 12 with *n* = 0.5) shows a considerably lower value of lgF, which may indicate its correctness. Also, according to the literature [35,36,37,58,68], the activation energy of the ethylenediamine elimination from the coordination sphere of transitional metals is in the range of 100–200 kJ/mol, which is consistent with the E values estimated according to the models 7 and 12 (*n* = 1).

Thus, the observed gas evolution during the thermal decomposition of [Ni(En)_3_](ClO_4_)_2_ is in complete agreement with known facts about the thermal decomposition of ethylenediamine complexes. It demonstrates the presence of the separate stage of elimination of ethylenediamine from the coordination sphere of nickel and the high-temperature exothermal stage, which is associated with the decomposition of perchlorate anions. Additionally, the molar ratio of ethylenediamine: oxygen in gas phase, taking their sensitivity coefficients into account, is estimated to be 1:1.25, which corresponds to the mass ratio of 60% to 40%.

In this way, based on the DMSTA data, we can conclude that the process of thermal decomposition of [Ni(En)_3_](ClO_4_)_2_ complex in an inert atmosphere contains at least two stages, accompanied by the release of ethylenediamine and perchlorate decomposition. So, in the next stage of modeling the DTG data obtained within the traditional non-isothermal thermal analysis at heating rate of 5 °C/min, the separating at least two stages and the contracting cylinder mechanism (model 7, Table 1) for the first stage of ethylenediamine elimination should be taken into consideration.

#### 3.2.2. Non-Isothermal Thermogravimetric Data

**Figure 4:**
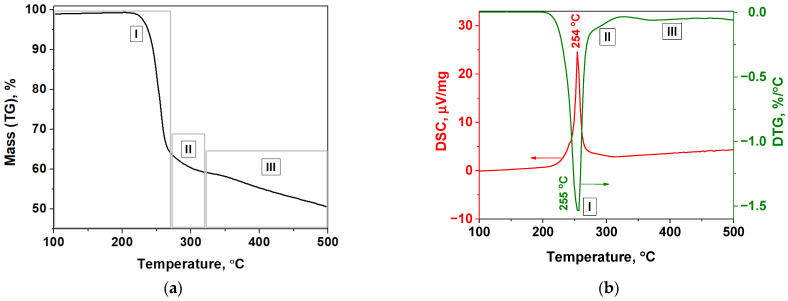
(**a**) Integral TG, (**b**) DTG and DSC curves according to the thermal analysis of [Ni(En)_3_](ClO_4_)_3_ decomposition in helium with β = 5 °C·min^−1^.

Thermogravimetric analysis shows integral and differential mass loss during heating, which, together with DMSTA results, makes it possible to investigate the reaction mechanism. During the thermogravimetric analysis of [Ni(En)_3_](ClO_4_)_2_ decomposition with the heating rate of 5 °C·min^−1^ (Figure 4) a fast weight loss (~35%) is observed in the temperature region of 210–270 °C (I) with the maximum on the DTG curve at 255 °C. This process is accompanied by an intense heat release (Figure 4b). Further, in the temperature region of 270–320 °C, another exothermic process is observed, which is characterized by a significantly lower mass loss rate (II). This process appears as a shoulder on the DTG curve in this temperature range. The total mass loss in these two regions was 40.4%.

**Figure 5:**
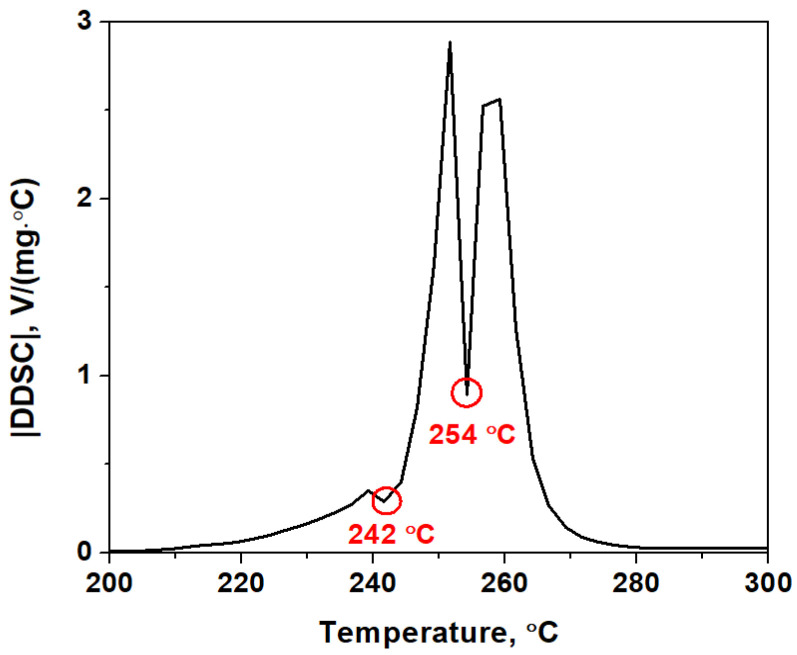
|DDSC| curve obtained at DSC data differentiating (Figure 4b).

According to the DMSTA, at least the elimination of one ethylenediamine molecule and the decomposition of perchlorate anions should be considered. Therefore, the observed mass loss in the stage I cannot be attributed to the endothermic process of elimination of all three ethylenediamine molecules from the coordination sphere of nickel (the theoretical mass loss of 41.1%) [34,35]. Also, the formation of Ni(En)_2_Cl_2_ due to elimination of one ethylenediamine molecule and decomposition of all perchlorate anions does not occur (mass loss of 43%) because the endothermic stages of ethylenediamine elimination, which are characteristic of Ni(En)_2_Cl_2_ thermolysis, are not observed in the DTG and DSC curves with further sample heating > 300 °C [34]. It should be noted that the DSC curve contains two inflection points in the temperature region of 205–270 °C, which are clearly visible when differentiating the DSC curve (Figure 5). Thus, together with the DMSTA results, this indicates that at least two overlapping stages occur in the temperature region I during the thermal decomposition of [Ni(En)_3_](ClO_4_)_2_.

Previously, the DMSTA results showed that the molar ratio of ethylenediamine: oxygen in the gas phase during the decomposition of [Ni(En)_3_](ClO_4_)_2_ was 1:1.25. Taking only these gaseous products into account, the calculated mass loss should be ~23%. Furthermore, the measured oxygen evolution during DMSTA is much lower than that which would be expected from the complete decomposition of perchlorate anions (4 moles). All these calculations show that the main weight loss (~35%), observed on the DTG curve in the temperature region of 205–270 °C (region I), is also associated with redox interactions between bound or free ethylenediamine and perchlorate anions or products of their decomposition, as discussed in [36]. It should also be noted that the intense process of thermal decomposition of [Ni(En)_3_](ClO_4_)_2_ coincides with the intense process of thermal decomposition of Ni(ClO_4_)_2_ (250 °C), which is accompanied by oxygen evolution [65].

The fast mass loss in the region I was modeled without the contribution of the high-temperature mass loss processes (II, III) characterized by significantly lower rate. The search for the kinetic parameters using the hybrid GA with the Coates-Redfern approximation (Equation (4)) was performed under the assumption that the process can be described by one or two limiting stages. When modeling the thermolysis process, all models were applied (1–11 and 12 with *n* from 1 to 3.5 with the step of 0.5 in Table 1).

The results show that the thermal decomposition of [Ni(En)_3_](ClO_4_)_2_ is described well as a single-stage process (Table S3). However, modeling it as a two-stage process improves the goodness of fit as indicated by the lower values of F (Table S4). At the same time, many combinations of the two-stage kinetic models tested in the DTG data modeling had similar values F (Table S4), but showed different kinetic parameters and mass loss fractions attributed to each stage (Tables S5 and S6). It was also noted that almost all of the acquired kinetic parameter sets form a linear dependence of E on lgA (Figure S4), which is widely known in the literature as kinetic compensation effect [11,19,41,69].

We believe that the unambiguity of the kinetic model selection during the modeling of the simulated DTG curve (described in Section 3.1) and the impossibility of choosing the correct kinetic models based only on the optimized values of F (Equation (6)) when modeling the real experimental data are due to the influence of different chemical and physical processes, as well as conditions of thermal analysis experiments [11].

**Figure 6:**
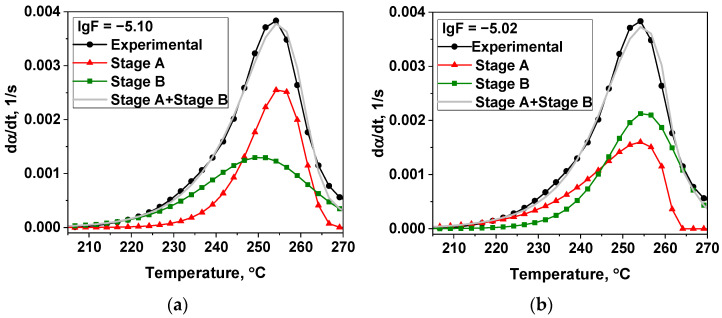
Models of thermal decomposition of [Ni(En)_3_](ClO_4_)_2_ in helium with β = 5 °C·min^−1^ (Table 4): (**a**) variant 1 and (**b**) variant 2.

Out of 136 kinetic model combinations used for the modeling of the two-stage process of thermal decomposition of the [Ni(En)_3_](ClO_4_)_2_ in region I (Tables S4–S6) only 9 of the most important results were chosen by the criteria based on the DMSTA results and literature: (1) one of the stages should describe the mass loss close to elimination of one ethylenediamine molecule (14 ± 4%) (Table S5), (2) activation energy of this stage should be in the 100–200 kJ/mol region (Table S6). Out of 9 models, 2 options with the best values of the objective function (lgF ≤ −5) were picked and presented in Table 4 and Figure 6.

The second variant of the modeling of the thermal decomposition (Table 4) better fits the DMSTA results discussed above, because, as in the DMSTA, the kinetic model 7 (contracting cylinder model in Table 1) describes the ethylenediamine elimination (stage A) more precisely, and the mass loss of 15.7% better corresponds to the evolution of one molecule of ethylenediamine (13.7%). Also, it should be noted that stage A in variant 2 begins before stage B (Figure 6b), which is in agreement with literature data for ethylenediamine complexes [34,35,36,37,59]. On the contrary, in variant 1 (Figure 6a) stage A begins shortly after stage B.

**Figure 7:**
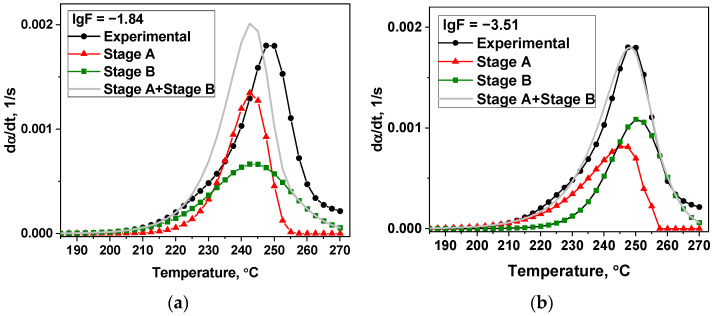
Prediction of DTG curve for [Ni(En)_3_](ClO_4_)_2_ decomposition obtained at a lower heating rate of 2.5 °C·min^−1^ using the models of Table 4: (**a**) variant 1 and (**b**) variant 2.

To test the proposed approach to modeling the thermal decomposition of [Ni(En)_3_](ClO_4_)_2_ using the data obtained at one heating rate of 5 °C·min^−1^, the model variants discussed in Table 4 were used to predict the experimental data at a lower heating rate of 2.5 °C·min^−1^. The original TG, DTG, and DSC curves are shown in Figure S5. It was also shown that the second variant predicts the DTG curve well (lgF = −3.51) (Figure 7b), while the first variant is unable to predict the results of the thermal analysis at the lower heating rate (lgF = −1.84) (Figure 7a). It additionally validates the obtained modeling results.

**Figure 8:**
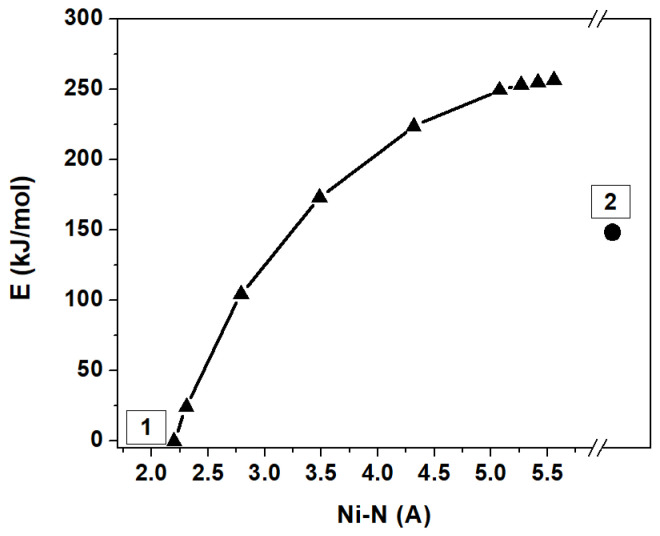
The profile of relative energy of the NEB-CI path for elongation of Ni−N bond during the elimination of one En molecule from [Ni(En)_3_]^2+^, where 1 is the initial [Ni(En)_3_]^2+^ state and 2 is fully optimized [Ni(En)_2_]^2+^ + En system.

The DFT calculations were carried out to verify the value of activation energy for the ethylenediamine elimination in the proposed model (stage A, variant 2, Table 4). Figure 8 shows the change in system energy that occurs as the Ni-N bonds elongate during the removal of one En molecule from [Ni(En)_3_]^2+^. It was shown that when the Ni-N distance increases, there is an increase in energy relative to the initial [Ni(En)_3_]^2+^ state. Unfortunately, the resulting relation does not allow determining the true geometry of the activated complex, but the vibrational frequency analysis confirmed the presence of only one imaginary frequency for the structure with Ni-N length of 5.57 Å, which may indicate the transition state. This fact permits us to estimate the transition state energy as 256 kJ/mol, which is comparable with E calculated for the first stage within model 2 (Table 4). The discrepancy between these two values of the activation energy may be explained by the high energy dependence on the used basis set, the absence of anions in the calculations, and the possibility of the existence of a lower-energy pathway with a different transition state.

The discrepancy between the mass fraction of the stage of ethylenediamine elimination during DMSTA (60%) and during thermogravimetric analysis (44%, stage A, variant 2, Table 4) can be explained by the contribution of the side processes. We believe that the higher gasification at stage B (56%, variant 2, Table 4) on the DTG curve (Figure 6b) compared to the mass fraction of released oxygen in DMSTA (40%) is associated with the release, in addition to oxygen, of other gaseous products of oxidation and degradation of ethylenediamine. This is consistent with the fact that the process of thermal decomposition of [Ni(En)_3_](ClO_4_)_2_ in the studied temperature range I (205–270 °C) cannot be explained by the formation of [Ni(En)_2_]Cl_2_ because characteristic stages of the thermolysis of this compound at higher temperatures are not detected [34]. At the same time, taking into account the total oxygen content in the composition of the initial complex, and the experimental fact of the release of molecular oxygen into the gas phase established by DMSTA, it can be argued that there is not enough “active oxygen” for the complete oxidation of ethylenediamine in the composition of the complex. We believe that there is a partial oxidation/degradation of ethylenediamine with the formation of both gaseous and solid organic products.

It is known that the decomposition of the perchlorate anions results in the formation of intermediates that are stronger oxidizers (ClO_3_^−^, ClO_2_^−^ and ClO^−^) [65,67], which can decompose further:

(9) $${\mathrm{ClO}}_{4-x}^{-}\;\to {\mathrm{ClO}}_{4-x-1}^{-}+\frac{1}{2}O_{2}\;\left(x=0\text{–}3\right)$$

The oxidizing ability of ClO_4_^−^ increases with the loss of oxygen [70]. We believe that it is these anions (${\mathrm{ClO}}_{4-x}^{-}$ x = 1–3) that predominantly interact with the ethylenediamine molecules:

(10) $${\mathrm{ClO}}_{4-x}^{-}+\;\mathrm{En}\;\to {\mathrm{Cl}}^{-}+\mathrm{gaseous}\;\mathrm{and}\;\mathrm{solid}\;\mathrm{products}\;\left(x=1\text{–}3\right)$$

Also, a reaction of free or bound ethylenediamine with gaseous oxygen released during perchlorate decomposition might occur:

(11) $$O_{2}+\;\mathrm{En}\;\to \mathrm{gaseous}\;\mathrm{and}\;\mathrm{solid}\;\mathrm{products}$$

All of these processes are consistent with the fact that the processes of redox reactions [29] and thermal decomposition of metal perchlorates [65,67] are characterized by high activation energies, which is observed when modeling stage B (Table 4). The subsequent high-temperature stages II and III (>270 °C) of slow mass loss can be attributed to the degradation of solid oxygen-containing products formed at stage I (Figure 4).

The suggested scheme of [Ni(En)_3_](ClO_4_)_2_ decomposition explains the overall low degree of its gasification upon reaching a temperature of 500 °C in helium (~50%) in comparison to the calculated value of its decomposition to NiO or Ni (83 or 87%, respectively).

Thus, the use of the hybrid GA for the modeling of the thermal analysis data in combination with the DMSTA results made it possible to divide the explosive-like process of the main mass loss during the thermal decomposition of [Ni(En)_3_](ClO_4_)_2_ into two stages and calculate their kinetic parameters.

## 4. Conclusions

In this work, it was shown that the use of the hybrid GA with the NM simplex algorithm when solving the nonlinear Coats–Redfern approximation (Equation (4)) leads to the correct results when describing simulated DTG curves for given parameters (2 stages, kinetic models, E, lgA, Table 2) with two overlapping stages in each run of the algorithm. Therefore, this approach was applied to model the thermal decomposition of [Ni(En)_3_](ClO_4_)_2_ complex in inert gas under non-isothermal conditions.

In the first step, DMSTA of the [Ni(En)_3_](ClO_4_)_2_ was carried out. It was shown that the decomposition of this compound should be described by at least two separate stages, accompanied by the release of ethylenediamine from the nickel coordination sphere and the evolution of oxygen during the decomposition of perchlorate anions. The molar ratio of ethylenediamine to oxygen in the released gases was 1:1.25. With the use of the hybrid GA, it was established that the stage of ethylenediamine evolution can be described by the contracting cylinder kinetic model with the activation energy of 122 kJ/mol. Probably, the obtained activation energy value may be underestimated due to the problem of the insufficient response time of the thermocouple under ultrafast heating conditions (~15–50 °C·s^−1^) of the DMSTA experiments.

In the second step, the thermal decomposition of [Ni(En)_3_](ClO_4_)_2_ was studied with the widely used thermogravimetric analysis with a heating rate of 5 °C·min^−1^. It was shown that the modeling of the DTG curve using the hybrid GA as two stages makes it possible to improve the accuracy of the mathematical description of the process in comparison to the description as a one-stage process. However, it was impossible to select only one reliable kinetic model based on the values of the objective function (F, Equation (6)) alone. To select right two-stage model, additional criteria were considered, which were derived from the data obtained by the dynamic mass-spectral thermal analysis (DMSTA) and analysis of literature data for the thermal decomposition of ethylenediamine complexes. As a result, only those models which met the elimination of one molecule of ethylenediamine from the coordination sphere of nickel were analyzed. For the first time, for the explosive-like thermal decomposition of [Ni(En)_3_](ClO_4_)_2_, a two-stage scheme was proposed. One of the stages describes ethylenediamine elimination with the activation energy of 192 kJ/mol. It should be noted that, as in the case of DMSTA results, this stage is also described by the contracting cylinder kinetic model. The obtained value of activation energy is comparable to the transition state energy estimated by DFT method.

The second stage includes the thermal decomposition of perchlorate anions, accompanied by the release of oxygen and the formation of stronger oxidizing agents ${\mathrm{ClO}}_{4-x}^{-}$ (x = 1–3), which instantly starts an explosive-like exothermic oxidation of ethylenediamine. This stage is described by n-order kinetic with *n* = 1.5 and has a higher activation energy of 351 kJ/mol, which is typical for various processes of oxidation of organic substances and decomposition of metal perchlorates.

Thus, it was shown that the hybrid GA can be used to extract kinetic parameters of the thermal decomposition process characterized by two overlapping stages. However, additional studies are needed to verify the proposed kinetic models by modeling. In our case, the combination of the methods such as DMSTA (β~15 °C·s^−1^), TG/DSC (β = 5 °C·min^−1^) and DFT was successfully applied. It was further shown that the proposed model predicts the DTG curve obtained at a lower heating rate of 2.5 °C·min^−1^ well, confirming the applicability of the applied modeling approach.

## Figures and Tables

**Table 1:** The solid-state reaction models.

	Model	Differential Form f(α)	Integral Form g(α)
Sigmoidal models			
1	Power law	2α^1/2^	α^1/2^
2		3α^2/3^	α^1/3^
3		4α^3/4^	α^1/4^
4	Avrami-Erofeev	2(1 − α)·[−ln(1 − α)]^1/2^	[−ln(1 − α)]^1/2^
5		3(1 – α)·[−ln(1 – α)]^2/3^	[−ln(1 − α)]^1/3^
6		4(1 – α)·[−ln(1 – α)]^3/4^	[−ln(1 − α)]^1/4^
Geometrical contraction models			
7	Contracting cylinder	2(1 − α)^1/2^	1 − (1 − α)^1/2^
8	Contracting sphere	3(1 − α)^2/3^	1 − (1 − α)^1/3^
Diffusion models			
9	1D Diffusion	1/(2α)	α^2^
10	Jander (3D Diffusion)	(3/2)·(1 − α)^2/3^/[1 − (1 − α)^1/3^]	[1 − (1 − α)^1/3^]^2^
11	Ginstling-Brounshtein (3D Diffusion)	(3/2)/[(1 − α)^−1/3^ − 1]	1 − (2α/3) − (1 − α)^2/3^
Reaction-order models (*n* = order)			
12	*n* = 1	1 − α	−ln(1 − α)
	*n* ≠ 1	(1 − α)*^n^*	[1 − (1 − α)^1−*n*^]/(1 − *n*)

**Table 2:** Comparison of kinetic parameters for simulated and predicted DTG curves.

	Stage	Kinetic Model	lgF	lgA	E, kJ/mol	Fraction
Simulated curve	1	10	-	30.00	240.00	0.5000
	2	8		32.00	250.00	0.5000
Best predicted curve	1	10	−10.82	30.01	240.09	0.5004
	2	8		32.01	250.07	0.4996
Second best predicted curve	1	12 (*n* = 3/4)	−8.18	13.99	117.63	0.4807
	2	8		31.90	249.35	0.5193

**Table 3:** Results of the modeling of the ethylenediamine with *m*/*z* = 30.

Model	lgF	lgF before 216 °C	lgA	E, kJ/mol
4	−2.07	−1.86	6.63	61
5	−2.07	−1.85	2.98	29
6	−2.07	−1.85	1.23	14
7	−2.06	−2.03	13.04	122
12 (*n* = 1)	−2.05	−1.86	18.78	171

**Table 4:** Two chosen results for the modeling of the main peak of the DTG curve (region I) during the thermal decomposition of [Ni(En)_3_](ClO_4_)_2_ in helium with β = 5 °C·min^−1^.

N	lgF	Stage	Model	lgA	E, kJ/mol	Fraction	Mass Loss, wt%
1	−5.10	A	5	9.67	121	0.50	17.8
		B	12 (*n* = 1.5)	21.02	231	0.50	17.7
2	−5.02	A	7	16.62	192	0.44	15.7
		B	12 (*n* = 1.5)	32.79	351	0.56	19.8

## Data Availability

Not applicable.

## Supplementary Materials

The following supporting information can be downloaded at: https://www.mdpi.com/article/10.3390/ma16010090/s1, Figure S1: ATR FTIR spectrum of synthesized [Ni(En)_3_](ClO_4_)_2_; Figure S2: Example of the surface of the functional (Equation (6)) for a single-stage simulated DTG curve (first order kinetics (Table 1), lgA = 25, E = 300 kJ/mol); Figure S3: Average determination coefficient R^2^ and average duration of single run of the algorithm for the modeling of the thermogravimetry data of [Ni(En)_3_](ClO_4_)_2_ decomposition as two-stage first order kinetics using the hybrid GA with the traditional γ_2_ = 1, hybrid GA with the γ_2_ proposed in this work and non-hybrid GA; Figure S4: Correlation between E and lgA for (a) stage A and (b) stage B; Figure S5: Thermal analysis data of decomposition of [Ni(En)_3_](ClO_4_)_2_ in He at lower heating rate of 2.5 °C·min^−1^; Table S1: Logarithms of the optimized values of the objective function (lgF) of all results of the modeling of the simulated curve (Table 2) using the hybrid GA; Table S2: Results of the modeling of the ethylenediamine (*m*/*z* = 30) evolution curve during the dynamic mass-spectral thermal analysis of [Ni(En)_3_](ClO_4_)_2_ using the hybrid GA; Table S3: Results of the modeling of the thermogravimetric analysis data of [Ni(En)_3_](ClO_4_)_2_ thermolysis (β = 5 °C·min^−1^) as a single-stage process using the hybrid GA; Table S4: Logarithms of the optimized values of the objective function (lgF) of all results of the modeling of the thermogravimetric analysis data of [Ni(En)_3_](ClO_4_)_2_ thermolysis (β = 5 °C·min^−1^) as a two-stage process using the hybrid GA; Table S5: Mass loss (%) attributed to the stage A for all results of the modeling of the thermogravimetric analysis data of [Ni(En)_3_](ClO_4_)_2_ thermolysis (β = 5 °C·min^−1^) as a two-stage process using the hybrid GA; Table S6: Activation energy (kJ/mol) of stage A for all results of the modeling of the thermogravimetric analysis data of [Ni(En)_3_](ClO_4_)_2_ thermolysis (β = 5 °C·min^−1^) as a two-stage process using the hybrid GA.
