# Supplementary material for: The Use of Hybrid Genetic Algorithm in the Kinetic Analysis of Thermal Decomposition of [Ni(C2H8N2)3](ClO4)2 with Overlapping Stages
Source: Materials (Basel). 2022 Dec 22;16(1):90. doi: 10.3390/ma16010090 (PMC9821161; doi:10.3390/ma16010090)
Supplement: Supplementary file 1 [file materials-16-00090-s001.zip › materials-2074612-supplementary.pdf]

Supplementary Materials

THE USE OF HYBRID GENERIC ALGORITHM IN THE KINETIC ANALYSIS OF THERMAL DECOMPOSITION OF $[Ni(C_2H_8N_2)_3](ClO_4)_2$ WITH OVERLAPPING STAGES

Kirill A. Dmitruk [1,2], Oxana V. Komova [1,4,*], Alexander A. Paletsky [1,3], Andrey G. Shmakov [3], Svetlana A. Mukha [1], Vladislav R. Butenko [1,2], Alena A. Pochtar [1,2], Olga V. Netskina [1,2]

[1] Boreskov Institute of Catalysis SB RAS, 5 Akademika Lavrentieva ave., 630090 Novosibirsk, Russia

[2] Novosibirsk State University, 1 Pirogova str., Novosibirsk, 630090, Russia

[3] Voevodsky Institute of Chemical Kinetics and Combustion SB RAS, 3 Institutskaya str., 630090 Novosibirsk, Russia

[4] Siberian Branch of Russian Academy of Sciences, 17 Akademika Lavrentieva ave., 630090 Novosibirsk, Russia

***** Correspondence: komova@catalysis.ru (O.V.K.) Tel.: +7-383-330-74-58

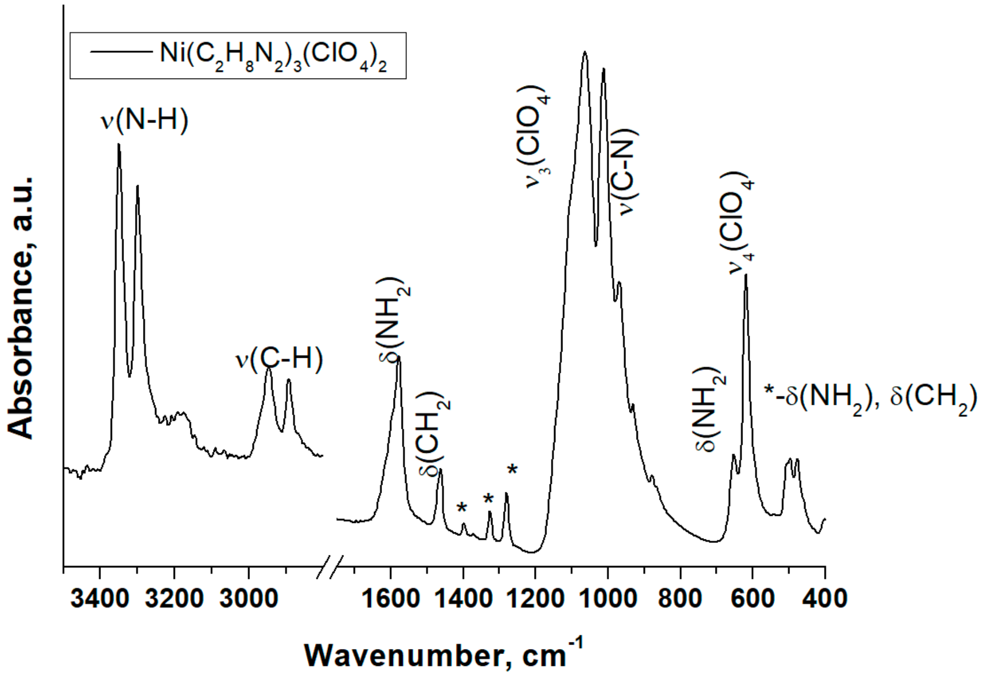

Figure S1. ATR FTIR spectrum of synthesized $[Ni(En)_3](ClO_4)_2$.

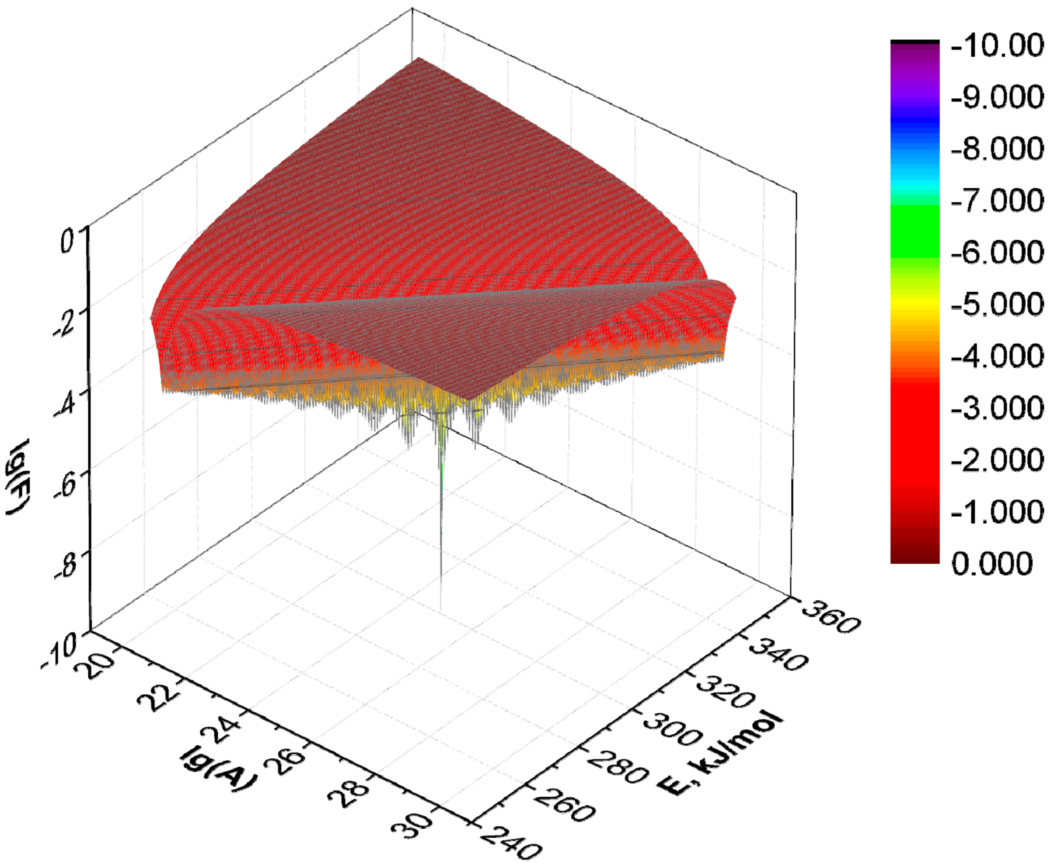

Figure S2. Example of the surface of the functional (Eq. 6) for a single-stage simulated DTG curve (first order kinetics (Table 1), lgA = 25, E = 300 kJ/mol).

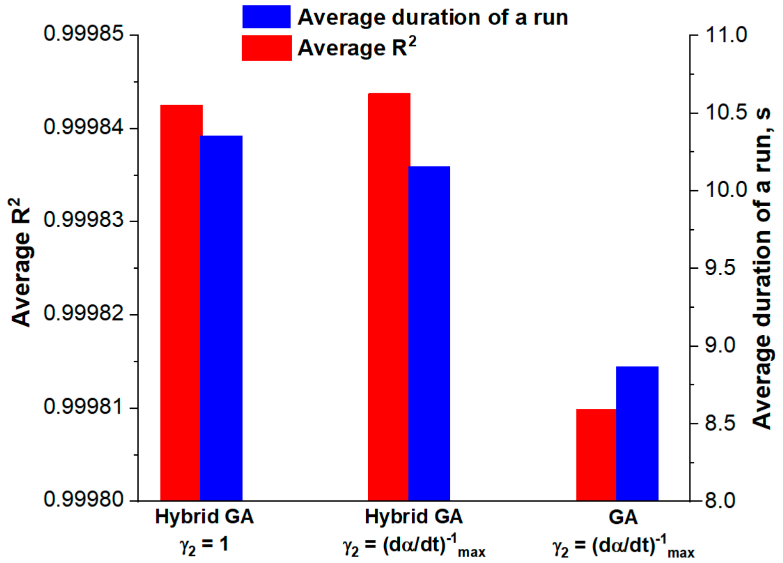

Figure S3. Average determination coefficient R^2 and average duration of single run of the algorithm for the modeling of the thermogravimetry data of $[Ni(En)_3](ClO_4)_2$ decomposition as two-stage first order kinetics process using the hybrid GA with the traditional γ_2=1, hybrid GA with the γ_2 proposed in this work and non-hybrid GA.

40 runs have been performed in each case.

Table S1. Logarithms of the optimized values of the objective function (lgF) of all results of the modeling of the simulated curve (Table 2) using the hybrid GA.

Model	1	2	3	4	5	6	7	8	9	10	11	n = 1/3	n = 3/4	n = 1	n = 1.5	n = 2	n =3
1	-3.04																
2	-3.04	-5.20															
3	-3.00	-5.09	-3.04														
4	-5.05	-4.84	-4.21	-3.22													
5	-5.04	-4.56	-4.66	-3.65	-4.34												
6	-4.72	-5.02	-4.06	-5.61	-5.63	-3.66											
7	-4.41	-3.18	-2.97	-5.26	-5.01	-5.84	-4.47										
8	-5.11	-4.19	-5.09	-5.20	-5.20	-6.08	-7.39	-6.47									
9	-5.23	-5.22	-3.93	-5.60	-5.05	-5.05	-5.71	-5.92	-4.09								
10	-5.53	-4.99	-5.01	-5.57	-5.57	-5.57	-5.50	-10.82	-4.98	-5.63							
11	-5.30	-5.30	-5.29	-5.85	-5.55	-5.32	-5.35	-6.05	-5.30	-3.41	-5.08						
n = 1/3	-3.71	-5.04	-4.28	-5.25	-5.72	-5.26	-4.37	-6.66	-5.55	-5.08	-5.34	-5.06					
n = 3/4	-4.77	-4.78	-4.78	-5.14	-5.59	-4.07	-4.90	-8.18	-5.60	-5.64	-5.62	-5.08	-4.70				
n = 1	-5.48	-5.49	-4.75	-5.58	-5.59	-5.22	-5.60	-4.50	-5.47	-5.56	-5.66	-5.60	-5.63	-5.17			
n = 1.5	-5.60	-4.21	-4.85	-5.60	-5.61	-5.61	-5.76	-7.17	-5.59	-5.57	-5.85	-5.71	-4.70	-5.47	-4.63		
n = 2	-5.39	-3.51	-3.51	-5.54	-5.55	-5.55	-5.55	-6.51	-5.38	-4.49	-5.54	-5.07	-4.83	-4.91	-5.54	-4.41	
n = 3	-3.34	-3.34	-3.34	-5.45	-5.46	-5.46	-5.51	-6.36	-5.26	-5.26	-5.48	-5.46	-4.71	-3.86	-5.45	-3.45	-3.08

Table S2. Results of the modeling of the ethylenediamine (m/z = 30) evolution curve during the dynamic mass-spectral thermal analysis of $[Ni(En)_3](ClO_4)_2$ using the hybrid GA.

Model	lgF	lgF before 216 °C	lgA	E, kJ/mol
4	-2.07	-1.86	6.63	61
5	-2.07	-1.85	2.98	29
6	-2.07	-1.85	1.23	14
7	-2.06	-2.03	13.04	122
12 (n = 1)	-2.05	-1.86	18.78	171
11	-2.03	-2.07	31.48	296
12 (n = 1.5)	-1.85	-1.69	23.88	216
12 (n = 2)	-1.69	-1.55	28.74	260
12 (n = 2.5)	-1.56	-1.45	33.43	302
8	-1.55	-1.55	7.61	75
12 (n = 3)	-1.46	-1.38	38.01	343
12 (n = 3.5)	-1.39	-1.31	42.49	383
9	-1.35	-1.39	16.48	153
1	-1.32	-1.34	1.00	13
10	-1.29	-1.20	21.87	210
2	-1.02	-1.13	1.00	13
3	-0.67	-0.79	1.00	14

Table S3. Results of the modeling of the thermogravimetric analysis data of $[Ni(En)_3](ClO_4)_2$ thermolysis (β=5 °C·min^{-1}) as a single-stage process using the hybrid GA.

Model	lgF	lgA	E, kJ/mol
1	-2.74	5.74	84
2	-2.70	2.54	53
3	-2.60	1.00	38
4	-4.37	10.18	126
5	-4.36	5.59	82
6	-4.35	3.27	59
7	-3.34	18.17	209
8	-2.95	18.98	219
9	-2.72	30.13	329
10	-3.69	42.66	460
11	-3.21	37.94	414
12 (n = 1)	-4.37	23.79	261
12 (n = 1.5)	-3.53	29.31	314
12 (n = 2)	-3.09	34.98	370
12 (n = 2.5)	-2.84	40.86	427
12 (n = 3)	-2.69	46.97	487
12 (n = 3.5)	-2.58	53.29	548

Table S4. Logarithms of the optimized values of the objective function (lgF) of all results of the modeling of the thermogravimetric analysis data of $[Ni(En)_3](ClO_4)_2$ thermolysis (β=5 °C·min^{-1}) as a two-stage process using the hybrid GA.

Model	1	2	3	4	5	6	7	8	9	10	11	n = 1	n = 1.5	n = 2	n = 2.5	n = 3	n = 3.5
1	-2.88																
2	-2.74	-2.70															
3	-2.40	-2.75	-2.64														
4	-4.80	-4.37	-4.63	-4.40													
5	-4.58	-4.57	-4.61	-4.37	-4.36												
6	-4.44	-4.56	-4.35	-4.40	-4.40	-4.39											
7	-2.91	-4.40	-4.24	-4.73	-4.46	-4.49	-4.79										
8	-3.24	-3.16	-3.16	-4.81	-4.56	-3.16	-4.56	-3.33									
9	-3.47	-3.26	-3.16	-4.77	-4.58	-4.57	-4.43	-3.51	-3.56								
10	-4.13	-4.10	-4.69	-5.01	-4.87	-4.85	-4.83	-4.13	-4.13	-4.98							
11	-3.82	-3.77	-3.98	-4.44	-4.93	-4.38	-4.79	-3.82	-3.74	-5.09	-4.06						
n = 1	-4.81	-4.76	-4.47	-4.47	-5.02	-4.40	-5.00	-4.40	-4.81	-4.87	-4.84	-5.02					
n = 1.5	-4.75	-4.08	-4.00	-5.10	-5.10	-4.12	-5.02	-4.70	-4.76	-5.11	-4.76	-5.10	-5.03				
n = 2	-4.44	-3.66	-3.77	-5.10	-5.09	-3.69	-4.88	-3.95	-4.45	-5.03	-4.77	-5.10	-5.08	-4.72			
n = 2.5	-4.24	-4.09	-3.31	-5.11	-5.10	-5.08	-4.69	-3.76	-4.25	-4.99	-4.65	-5.11	-5.04	-4.54	-4.20		
n = 3	-4.12	-3.97	-3.35	-5.12	-5.11	-5.08	-4.59	-3.65	-4.13	-4.98	-4.60	-5.12	-4.99	-4.43	-4.08	-3.88	
n = 3.5	-4.04	-3.89	-2.92	-5.12	-5.12	-3.24	-4.52	-3.36	-4.04	-4.96	-4.55	-5.13	-4.95	-4.35	-4.01	-3.19	-3.67

Table S5. Mass loss (%) attributed to the stage A for all results of the modeling of the thermogravimetric analysis data of $[Ni(En)_3](ClO_4)_2$ thermolysis (β=5 °C·min^{-1}) as a two-stage process using the hybrid GA.

Model	1	2	3	4	5	6	7	8	9	10	11	n = 1	n = 1.5	n = 2	n = 2.5	n = 3	n = 3.5
1	9.6%																
2	35.5%	35.2%															
3	12.1%	33.4%	33.9%														
4	5.1%	35.5%	3.6%	35.5%													
5	3.9%	3.5%	33.3%	35.5%	35.5%												
6	35.1%	3.5%	35.5%	35.5%	35.5%	35.2%											
7	15.3%	7.5%	32.7%	29.3%	0.5%	27.1%	23.7%										
8	20.0%	32.5%	32.5%	3.3%	34.3%	32.5%	31.5%	20.1%									
9	16.4%	20.0%	18.1%	32.0%	31.6%	31.5%	21.4%	18.9%	14.0%								
10	6.8%	6.2%	33.4%	9.2%	26.0%	21.3%	21.9%	5.9%	28.7%	2.2%							
11	11.0%	10.3%	32.6%	34.8%	23.2%	7.1%	24.1%	9.7%	20.2%	12.8%	28.1%						
n = 1	5.1%	3.9%	35.1%	35.0%	9.8%	35.5%	15.6%	0.3%	30.4%	2.2%	6.2%	25.7%					
n = 1.5	8.7%	4.8%	3.8%	17.8%	17.8%	31.8%	15.7%	3.7%	8.8%	12.7%	11.6%	17.7%	6.2%				
n = 2	13.0%	8.4%	12.8%	23.5%	23.4%	5.8%	19.5%	10.4%	13.1%	12.5%	18.1%	12.1%	11.8%	13.7%			
n = 2.5	15.7%	14.8%	3.0%	25.7%	25.7%	25.6%	22.7%	13.9%	15.7%	25.1%	20.3%	9.8%	8.9%	11.6%	14.2%		
n = 3	17.5%	16.6%	17.1%	27.0%	26.9%	27.6%	24.3%	16.3%	17.5%	26.4%	22.1%	8.6%	7.7%	10.3%	12.6%	14.3%	
n = 3.5	18.8%	18.0%	8.2%	27.7%	27.7%	9.6%	25.4%	20.3%	18.8%	27.3%	23.3%	7.8%	28.5%	9.5%	11.6%	32.0%	14.3%

Table S6. Activation energy (kJ/mol) of stage A for all results of the modeling of the thermogravimetric analysis data of $[Ni(En)_3](ClO_4)_2$ thermolysis (β=5 °C·min^{-1}) as a two-stage process using the hybrid GA.

	1	2	3	4	5	6	7	8	9	10	11	n = 1	n = 1.5	n = 2	n = 2.5	n = 3	n = 3.5
**1**	92																
**2**	84	54															
**3**	38	51	39														
**4**	101	126	38	126													
**5**	68	40	78	126	82												
**6**	61	39	59	126	82	60											
**7**	229	77	227	233	73	69	230										
**8**	84	242	242	63	79	242	230	188									
**9**	109	58	48	120	84	61	259	274	371								
**10**	119	80	489	180	99	77	231	370	443	336							
**11**	112	74	449	131	83	185	231	352	401	366	444						
**n = 1**	100	79	268	130	134	261	228	177	252	192	213	221					
**n = 1.5**	57	164	149	186	121	297	192	226	251	459	329	231	298				
**n = 2**	62	130	38	170	111	300	194	154	273	436	379	236	287	328			
**n = 2.5**	65	39	50	165	107	79	204	168	286	452	376	251	342	388	399		
**n = 3**	67	40	38	162	105	75	207	178	294	455	383	271	407	457	468	471	
**n = 3.5**	69	41	174	160	104	269	210	301	300	458	388	295	414	534	543	585	545

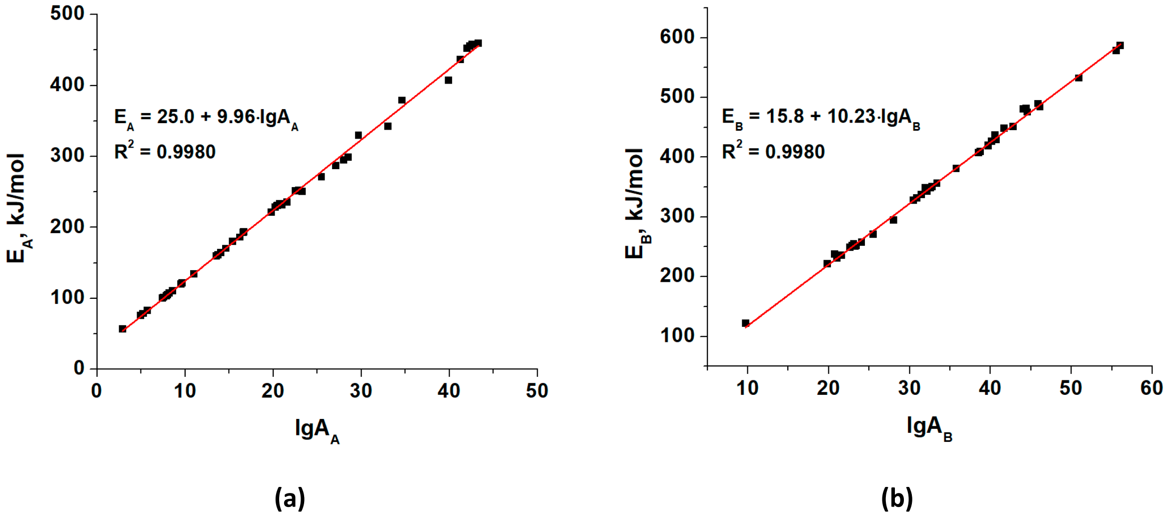

Figure S4. Correlation between E and lgA for (a) stage A and (b) stage B.

Only results with the determination coefficients R^2 higher than 0.9999 were used in this figure.

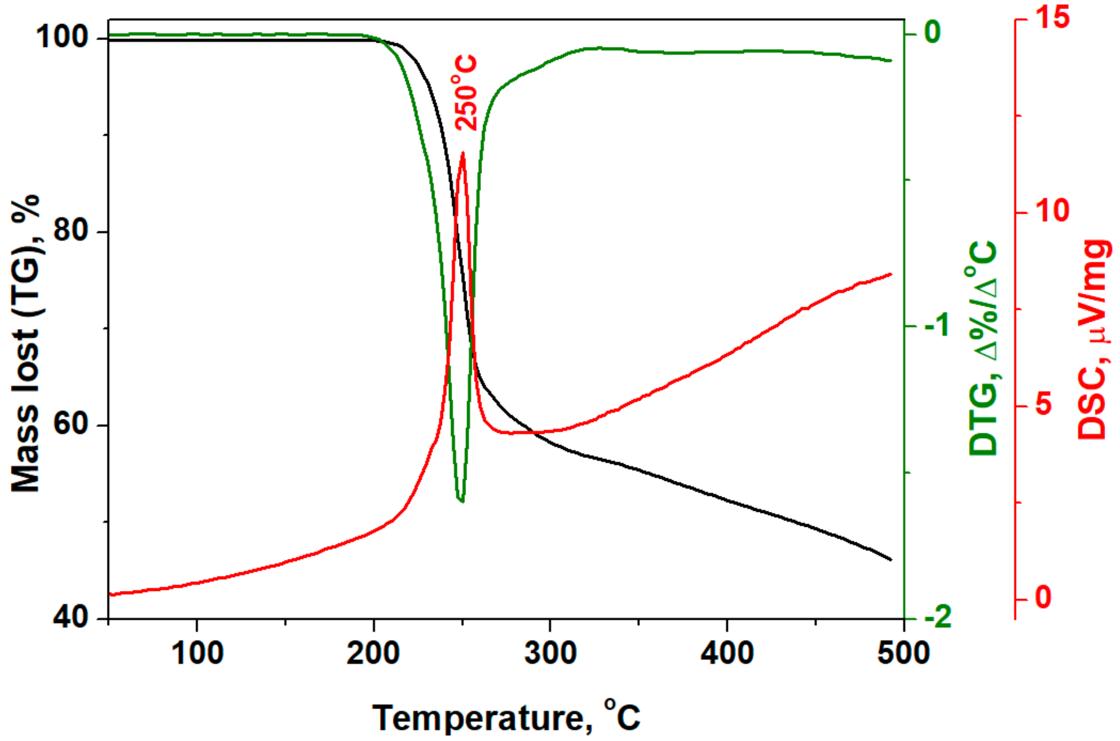

Figure S5. Thermal analysis data of decomposition of $[Ni(En)_3](ClO_4)_2$ in He at lower heating rate of 2.5 °C·min^{-1}.
